# Supplementary material for: ESCRT Machinery in HBV Life Cycle: Dual Roles in Autophagy and Membrane Dynamics for Viral Pathogenesis
Source: Cells. 2025 Apr 16;14(8):603. doi: 10.3390/cells14080603 (PMC12025488; doi:10.3390/cells14080603)
Supplement: Supplementary file 1 [file cells-14-00603-s001.zip › cells-3479178-supplementary.pdf]

Supplementary

Target				Intracellular					Extracellular				Colocalization	Reference
Target		Method	Model	RNA	pgRNA	DNA	Capsid	HBsAg	DNA	Capsid	Virion	HBsAg		
ESCRT-0	HRS/ HGS	siRNA	HepG2			↓	↓			↓	↓		HBc co-localizes with HGS near the cell periphery.	[58]
		Overexpression	HepG2	↓		↓	↓							
			Huh7	↓		↓	↓			↑	↓	↓		
			Qs21				↓			_	↓	↓		
			mouse			↓	↓		↓	_	↓	↓		
	STAM2	siRNA	HepG2, Huh7			↓	↓				↓			
		Overexpression	Huh7							_	↑	↑		
ESCRT-I	TSG101	siRNA	Huh7				_			↑				[56]
											↑			[57]
			HepG2-NTCP and HepAD38	_	_	↑	↑		↓		↓	↓	TSG101 VFND motif recongnizes HBc K96 and PPAY motif.	[59]
		shRNA	mouse						↓			↓		
	VPS28	siRNA	Huh7				_			↑				[56]
		siRNA	Huh7								↑			[57]
ESCRT-II	EAP20	siRNA	Huh7								↓			[57]
	EAP30	siRNA	Huh7		↓	↓	↓	_			↓			
	EAP45	siRNA	Huh7		↓	↓	↓	_			↓			
ESCRT-III	CHMP3	DN	Huh7				↓			↑				[56]
	CHMP3	DN	Huh7									↓		[55]
	CHMP3	DN	Huh7			↓	↓		↓		↓	_		[54]
	CHMP4B	DN	Huh7			↓	↓		↓		↓	_		
	CHMP4C	DN	Huh7			↓	↓	↓	↓		↓	_		
VPS4	VPS4	DN (E228Q,K173Q)	Huh7, HepG2			↓					↓			16920176
	VPS4A	DN (E228Q)				↓	↓	↓	↓		↓	_		[54]
		DN (E228Q)	Huh7									↓		[55]
		DN (E228Q)	Huh7				↓			↑				[56]
	VPS4B	DN (E235Q)				↓	↓		↓		↓			[54]
		DN (E235Q)	Huh7									↓		[55]
Alix		DN (bro)	HepG2							↓			HBcAg colocalizes with the Bro1 domain of Alix.	[60]
		DN (bro)	Huh7	↓					↓					[56]
		siRNA	Huh7	_					_	↓				
		siRNA	HepG2							↓				[60]
		WT	Huh7	↓					↓	↑				[56]
		WT	HepG2	_	_					↑				[60]

Table S1. Summary of ESCRT Machinery's Effects on HBV Transcription, Replication, and Secretion. This Figure outlines the observed effects of individual ESCRT proteins on various aspects of HBV biology, including intracellular and extracellular HBV RNA, pgRNA, DNA, HBsAg, capsid, and virion levels. The outcomes are categorized: "↑" denotes an increase and is highlighted in pink; "↓" denotes a decrease and is highlighted in blue; "-" denotes no changes and is highlighted in gray; white color without marks denotes no data reported. Experimental conditions, such as the use of siRNA, wild-type (WT), or dominant-negative (DN) mutant proteins, are also specified.
